# The impact of health insurance on self-protection of Chinese rural residents

**DOI:** 10.3389/fpubh.2022.874619

**Published:** 2022-09-16

**Authors:** Yao Li, Lei Li, Junxia Liu

**Affiliations:** ^1^School of Public Administration, Zhongnan University of Economics and Law, Wuhan, China; ^2^School of Economics, Zhongnan University of Economics and Law, Wuhan, China

**Keywords:** health insurance, ex ante moral hazard, self-protection, New Rural Cooperative Medical Scheme, Chinese rural residents

## Abstract

**Purpose:**

Health insurance lowers the price of medical services, which reduces the insured's demand for self-protection (such as, live a healthy lifestyle or invest in disease prevention) that could help reduce the probability of getting sick, then ex ante moral hazard happens. The purpose of this study is to examine the impact and its heterogeneity of health insurance on the self-protection of Chinese rural residents.

**Method:**

This study firstly builds a theoretical model of health insurance and self-protection. Then, based on the data from the 2004 to 2015 China Health and Nutrition Survey (CHNS), we adopt ordinary least squares model (OLS), probit model, and instrumental variable (IV) method to empirically investigate the impact of health insurance on Chinese rural residents' self-protection.

**Results:**

After addressing the endogeneity problem, the study finds that participating in health insurance exerts a significant negative impact on the demand for self-protection. Specifically, health insurance participation not only increases residents' tendency toward drinking liquor by 3.4%, and that of having general obesity by 3.7%, but also reduces residents' preventive medical expenditure (PME) by 1.057%, increasing Body Mass Index (BMI) by 0.784 kg/m^2^. Further analysis shows that there is heterogeneity between groups, as health insurance participation reduces PME of people who are female, younger, and high-educated, and increases the tendency toward drinking liquor of people who are younger and low-educated.

**Conclusions:**

To reduce the impact of ex ante moral hazard induced by health insurance, our findings suggest that it is necessary to improve the disease prevention function of health insurance and introduce a risk adjustment mechanism into the premium or co-payment design of health insurance.

## Introduction

In order to improve health insurance coverage and narrow the gap in health care usage between urban and rural areas, Chinese government launched the New Rural Cooperative Medical Scheme (NRCMS) in 2003 (1). Thanks to joint efforts of central and local governments, the enrollment of NRCMS steadily increased and had exceeded 95% in 2010, basically achieving the full coverage of Chinese rural population (2). According to annual reports of China National Health Services Survey, the actual hospital reimbursement rate of NRCMS has increased from 26.6% in 2008 to 45.4% in 2018. The medical needs of rural residents have been released to a certain extent due to NRCMS's fast expansion. However, while health insurance improves the accessibility of medical services by diversifying risks, it may cause some efficiency losses, such as moral hazard.

Theoretically, as health insurance lowers the marginal price of health services faced by the insured, two changes could happen in enrollees' health behaviors: for one thing, people may reduce their self-protection (e.g., live a healthy lifestyle; invest in disease prevention), which is the so-called “ex ante moral hazard” (3, 4); for another, people may over-consume medical care after getting sick, which can be termed “ex post moral hazard” (5, 6). Since Arrow introduced the concept of moral hazard to the field of health insurance, ex post moral hazard has been an important research topic (7). Compared to tremendous studies on ex post moral hazard (8–13), little attention has been paid to ex ante moral hazard. In fact, if there is no effective control of ex ante moral hazard, it will not only cause serious long-term health damage for the insured (14), but also lead to social welfare losses through the negative externality generated by risk-taking behavior of the insured (for instance, smoking and alcoholism). It was not until Ehrlich and Becker built a theoretical model of ex ante moral hazard based on health production function that ex ante moral hazard induced by health insurance enjoyed widely discussion.

According to Ehrlich and Becker (3), by decreasing the utility difference between healthy state and sick state, health insurance reduces the marginal gain of self-protection, which leads to a decline in the marginal productivity of self-protection, then the decline of the demand for self-protection. In other words, ex ante moral hazard induced by health insurance can be summarized as a negative correlation between health insurance and self-protection. However, the empirical evidence has been mixed.

Some scholars believe that health insurance reduces the demand for self-protection of the insured. For instance, using the data from America and structural equation modeling which included health insurance choices and four lifestyles, Stanciole found that health insurance significantly increased the probability of excessive smoking, lack of exercise, and obesity (15). Based on comprises longitudinal administrative data on employees in Brazil from 2004 to 2008, Maia et al. provided evidence in another way, and their study showed that in the last 2 months before leaving private health insurance, enrollees took more medical visits and diagnostic tests (16). Further studies pointed out that ex ante moral hazard induced by health insurance could be more prevalent in certain groups, such as elderly men and diabetic patients (17, 18). In addition, people covered by public insurance tended to face a higher risk of obesity than those covered by private insurance (19). Similar evidence has been found in Ghana and Mexico (20, 21).

In contrast, some scholars draw a different view that health insurance does not influence the demand for self-protection of the insured. For instance, using the data from the National Health Interview Survey (NHIS) in US from 2006 to 2016, Lipton found that health insurance exerted no substantial effects on the probability of overweight and obesity in adults with diabetes (22). Courbage and Coulon used British Household Panel Survey data and instrumental variable (IV) method to examine the impact of private health insurance on individual health behavior (23). The authors found that private insurance coverage not only increased one's propensity toward exercising and breast cancer screening, but also reduced the probability of smoking. Similarly, based on the data from the 2017 behavioral risk factor surveillance system (BRFSS) in US, Zhao et al. found that participation in health insurance would decrease people's probability of smoking and increase that of exercising (24). Using BRFSS from 2001 to 2016, McInerney and Meiselbach found that health insurance expansion contributed to the decline of Body Mass Index (BMI) among the severely obese (25). Some studies showed that before and after participating in health insurance, there were no differences in enrollees' self-protection behaviors, such as smoking, drinking, and utilization of diabetes preventive services (9, 26–28).

Recently, whether health insurance has substantial effects on the demand for self-protection in China attracts some attention. However, empirical conclusions fail to reach a consensus. For urban residents, Xie et al. found that participation in health insurance not only increased an individual's propensity toward exercising and physical examination, but also decreased their propensity toward smoking and drinking (29). When it turns to urban employee, Wang and Gong argued that after getting enrolled in the Urban Employee Medical Insurance, the insured tended to reduce their monthly alcohol consumption (30). For rural residents, health insurance could lead to the increase of unhealthy behavior, such as smoking, heavy drinking, and eating high-calorie food (31, 32). Some scholars have extended the research to specific groups. For rural residents beyond the age of 60, using 2005–2008 Chinese Longitudinal Healthy Longevity Survey data and PSM-DID model, Fu et al. found that after participation in health insurance, these residents tended to smoke and drink more, and exercise less (33). For rural male residents, on the basis of data from 2000 to 2009 China Health and Nutrition Survey (CHNS), Yu and Zhu found that health insurance reduced their propensity toward smoking and drinking (34).

The divergence of the above conclusions can be explained from following aspects. On one hand, as health insurance programs in different countries have different designs for health interventions, their impacts on self-protection could be different as well. For example, health insurance in some countries covers both disease treatment services and some self-protection, including smoking cessation, cancer screening, and other disease prevention items, while in some countries, health insurance only limits to disease treatment services (35). On the other hand, there is heterogeneity between groups. People of different gender, age, and education level have different demand for self-protection, which could also lead to inconsistent conclusions.

From the above literature review, the existence of ex ante moral hazard in health insurance has been well-discussed in developed countries. However, this topic has not yet attracted widespread attention from scholars in China and there are following points to improve on: (i) In terms of research content, the mechanism of how health insurance affects self-protection has not been theoretically demonstrated. (ii) In terms of research sample, previous studies mainly used survey data before 2011, the conclusions based on which have limited enlightenment on the current reform of China's health insurance system. (iii) In terms of empirical design, existing research only used several unhealthy behavior (such as smoking, drinking) as the proxy variables for self-protection, which could not comprehensively describe ex ante moral hazard induced by health insurance.

Therefore, firstly, this study builds a theoretical model of how health insurance affects self-protection. Secondly, based on the latest five-round data of the 2004–2015 CHNS, this paper selects six indicators from three aspects of healthy lifestyle, preventive medical usage, and obesity risk to measure self-protection behaviors of the insured, and adopts ordinary least squares model (OLS), probit model, and IV method to identify the effect of health insurance participation on Chinese rural residents' self-protection. In addition, we also conduct a heterogeneity analysis (including gender, age, and education level) to investigate the influences of health insurance participation among different groups.

The marginal contributions of this paper are as follows: (i) This study establishes a theoretical model of health insurance and self-protection, and proves that participation in health insurance would decrease the insured's demand for self-protection through reducing the marginal gain of self-protection. (ii) The latest five-round data of CHNS is used in this study, which is the most representative and newest data for studies to examine the relationship between health insurance and self-protection. (iii) Comparing with existing empirical studies, this paper applies more comprehensive indicators to measure self-protection behaviors. For instance, we adopt preventive medical expenditure (PME) as the proxy for self-protection, which is a more accurate indicator of self-protection but ignored by most studies.

## Theoretical model

Drawing on the analytical framework of Ehrlich and Becker (3), this study constructs a theoretical model of health insurance and self-protection as follows. It is assumed that: (i) The marginal utility of income is strictly declining, and this properties of the utility function can be expressed as: U' > 0, U” <0. (ii) An individual faces only two states of state 0 (sick) and state 1 (healthy) with probabilities P and 1 – P, respectively, and the corresponding actual incomes are I_0_ and I_1_. (iii) The expenditure on self-protection could reduce the probability of getting sick: P = P(p^e^, r) and ∂P/∂r = P'(r) ≤ 0, where r is the expenditure on self-protection, p^e^ is the endowed probability of getting sick, P is the actual probability of getting sick.

When a rational consumer is not covered by health insurance, the optimal expenditure on self-protection should maximize:


(1) $$\begin{array}{l} {\text{U}}^{*}=\left[1-\text{P}\left({\text{p}}^{\text{e}},\text{r}\right)\right]\text{U}\left({\text{I}}_{1}-\text{ r}\right)+\text{P}\left({\text{p}}^{\text{e}},\text{r}\right)\text{U}\left({\text{I}}_{0}-\text{r}\right)\; \end{array}$$

The first-order optimal condition is:


(2) $$\begin{array}{l} -{\text{P}}^{\text{′}}\left({\text{r}}^{0}\right)\left[\text{U}\left({\text{I}}_{1}-\text{ r}\right)-\text{ U}\left({\text{I}}_{0}-\text{r}\right)\right]=\left[1-\text{P}\left({\text{r}}^{0}\right)\right]{\text{U}}_{1}^{\text{′}}\\ \;+\text{P}\left({\text{r}}^{0}\right){\text{U}}_{0}^{\text{′}} \end{array}$$

In equilibrium, the term on the left represents the marginal gain from the decline in P, which is caused by the expenditure on self-protection; that on the right represents the marginal cost that is generated by the reduction in incomes of different states. In addition, r^0^ is the optimal expenditure on self-protection, while U(I_1_ − r) is the utility in state 1, U(I_0_ − r) is the utility in state 0.

When the individual gets insured, function (1) can be rewritten as:


(3) $$\begin{array}{l} {\text{U}}^{*}=[1-\text{P}({\text{p}}^{\text{e}},\text{r})]\text{U}({\text{I}}_{1}-\text{r}-\text{c})+\text{P}({\text{p}}^{\text{e}},\text{r})\text{U}({\text{I}}_{0}\\ \;-\text{r}-\text{c}+\text{s} \end{array}$$

In equilibrium, c represents the expenditure on health insurance, and s represents the economic compensation that the individual could get from health insurance after getting sick.

Accordingly, the value of r would be chosen to maximize the expected utility function:


(4) $$\begin{array}{l} -{\text{P}}^{\prime }({\text{r}}^{*})[\text{U}({\text{I}}_{\text{1}}-\;{\text{r}}^{*}-\text{ c})-\text{U}({\text{I}}_{\text{0}}-{\text{r}}^{*}-\text{ c}+\text{s})]\\ \text{ =}[\text{1}-\text{P}({\text{r}}^{*})]{{\text{U}}^{\prime }}_{\text{1}}({\text{I}}_{\text{1}}-{\text{r}}^{*}-\text{c})\\ \;+\text{P}({\text{r}}^{*}){{\text{U}}^{\prime }}_{\text{0}}({\text{I}}_{\text{0}}-{\text{r}}^{*}-\text{c}+\text{s}) \end{array}$$

In Equation (4), r^*^ is the optimal expenditure on self-protection when health insurance is available. Similarly, terms on the left and right sides of the equation represent, respectively, the marginal gain and the marginal cost when health insurance is available. Specifically, the left side shows that participating in health insurance will reduce the utility in state 1 (through paying the premium c) and increase the utility in state 0 (through getting an economic compensation s which is greater than c). Due to the decline of the difference between the utilities in different states, the marginal gain of the expenditure on self-protection decreases.

According to the principle of actuarial fairness, the price of health insurance is negatively related to the probability of getting sick, and combining the fact that ∂P/∂r = P'(r) ≤ 0, we get:


(5) $$\begin{array}{l} \text{∂c}/\text{∂r}={\text{c}}^{\text{′}}\left(\text{r}\right)<0 \end{array}$$

In reality, the premium paid by the individual who gets enrolled in NRCMS is determined by the policy, which means that the price of health insurance is independent of personal expenditure on self-protection. Considering that, we obtain:


(6) $$\begin{array}{l} \text{∂c}/\text{∂r}={\text{c}}^{\text{′}}\left(\text{r}\right)=0 \end{array}$$

By Equations (4) and (6), there will be a substitutional relation between health insurance and self-protection. A rational individual will reduce the expenditure on self-protection to maximize expected utility. Then, the optimal expenditure on self-protection, r^*^, can be smaller than the original value, r^0^, when health insurance is not available. In another words, participating in health insurance will lead to the decline of expenditure on self-protection.

## Methods

### Data

The data used in this study comes from CHNS, a project conducted by the Carolina Population Center at the University of North Carolina and the National Institute for Nutrition and Health at the Chinese Center for Disease Control and Prevention. The first survey was carried out in 1989 and follow-up surveys were conducted in 1991, 1993, 1997, 2000, 2004, 2006, 2009, 2011, and 2015. A multi-stage random cluster process was used by CHNS to draw a nationally representative sample. This survey collects comprehensive information related to rural residents' demographic characteristics, health behaviors and health insurance, which provides the empirical basis for our study. It is worth noting that CHARLS and CLHLS can also be used to examine the existence of ex ante moral hazard among China rural residents, and these two datasets have been updated to 2018. This paper selects CHNS as the data source for following reasons. On the one hand, while CHARLS only includes respondents aged 45 and above and CLHLS mainly surveys the elderly around 80 years old, the respondents of CHNS include rural residents of all ages, which can provide a more complete picture for assessing the impact of China's health insurance. On the other hand, among these datasets, only CHNS provides information on PME, which is taken as an important explained variable in this study. As NRCMS was launched in 2003, we restrict our sample to the rural respondents to the latest five waves of CHNS data (2004, 2006, 2009, 2011, and 2015). According to the purpose of this paper, observations under age 18 are dropped as these non-adult children's health behaviors tend to be influenced by their parents. Observations with other types of health insurance or missing information on key independent variables are deleted as well. Finally, 19,243 valid samples are obtained, among which 13,243 are covered by NRCMS.

### Variables

#### Dependent variables

The dependent variables in this study are self-protection behaviors. Following previous studies (21, 27, 31, 34) and considering the availability of data, we measure rural residents' self-protection behaviors from three aspects: healthy lifestyle, preventive medical usage, and obesity risk. Specifically, the behavioral indicators of healthy lifestyle contain smoking, drinking, and sedentary. Smoking which is constructed based on the question “Do you still smoke cigarettes now,” is a dummy variable representing whether the respondent has a habit of smoking cigarettes. Drinking which is constructed based on the question “Do you drink liquor,” is also a dummy variable representing whether the respondent has a habit of drinking liquor. Sedentary represents the daily sitting time (h) of the respondent, which is measured by following questions: (i) “Do you participate in following activities such as watching TV, surfing the internet, participating in chat rooms including QQ and WeChat, playing computer/smartphone games”; (ii) “How much time do you spend during a typical day?” Preventive medical expenditure is selected to reflect the usage of preventive medical care, which corresponds to the questions as “During the past 4 weeks, did you receive any preventive health service, such as health examination, eye examination, blood test, blood pressure screening, tumor screening?” and “How much did this service cost?” The behavioral indicators of obesity risk contain BMI and Obesity. Body Mass Index is calculated by dividing the weight (kg) by the squared height (m^2^) of each respondent. According to the criteria of weight for adults in Health Industry Standards of the People's Republic of China, the respondent is defined as having general obesity if the BMI ≥ 28.0 (32).

#### Independent variable

The independent variable of this study is a dummy whether the respondent is covered by health insurance in the survey year. We assign the health insurance variable as 0 and 1, with 0 representing that the respondent gets insured, and 1 representing that the respondent doesn't get insured. Since the type of health insurance covered in rural China is NRCMS, we can identify the respondent's health insurance status based on the question “Do you have participated in NRCMS?” If the answer is “yes,” the respondent can be defined as an health insurance enrollee.

#### Control variables

Based on existing research (31–36), we select respondents' demographic characteristics (age, gender, ethnicity, marital status), health status, socioeconomic characteristics (education, annual income, working), and the year of interview as control variables. The PME and annual income is measured in 2015 Yuan. The definitions of variables mentioned above are introduced in Table 1.

**Table 1:** Variables.

**Variable**	**Definition**
**Independent variable**	
Health insurance	Enrolled in NRCMS, yes = 1, no = 0
**Dependent variables**	
Smoking	Currently smoking, yes = 1, no = 0
Drinking	Drinking liquor, yes = 1, no = 0
Sedentary	Sedentary time per day (h)
PME	Amount of preventive medical expenditure in the past 4 weeks (Yuan) (take the logarithm)
BMI	Weight in kilograms divided by the squared height in meters (m)
Obesity	BMI ≥ 28, yes = 1, no = 0
**Control variables**	
Age	Age of respondents
Age^2^/100	Age^2^/100
Gender	Male = 1, Female = 0
Ethnic	Ethnicity, Han = 1, other = 0
Marriage	Marital status, married = 1, other = 0
Education	Primary school and below = 1, junior high school = 2, Senior high school and above = 3
Health status	Got sick in the past 4 weeks, yes = 1, no = 0
Income	Annual income last year (Yuan) (take the logarithm)
Working	Presently working, yes = 1, no = 0

### Models

#### Benchmark model

The following benchmark models are used to estimate the impact of health insurance on self-protection behaviors:


(1) $$\begin{array}{l} y_{it}=\alpha_{0}+\alpha_{1}insurance_{it}+\alpha_{2}X_{it}+\varepsilon_{it} \end{array}$$

(2) $$\begin{array}{l} \text{P}\left(y_{it}=1|insurance,X\right)=\text{P}\left(y_{it}^{{}^{*}}>0|insurance,X\right)\\ \;=G\left(\alpha_{1}insurance_{it}+\alpha_{2}X_{it}+\varepsilon_{it}\right) \end{array}$$

Among them, *y*_*it*_ denotes the self-protection behaviors of individual *i* in year *t*, which includes six indicators from healthy lifestyle, preventive medical usage and obesity risk. *Insurance*_*it*_ is a dummy variable indicating whether individual *i* gets covered by health insurance in year *t*. *X*_*it*_ contains a series of control variables. ε_*it*_ is the error term. In model (2), G is the cumulative distribution function of random error ε_*it*_ which follows the standard normal distribution. When *y*_*it*_ is a continuous variable (sedentary, PME, and BMI), we adopt OLS to estimate model (1). When *y*_*it*_ is a binary variable (smoking, drinking, and obesity), we use probit model which can avoid the problem of natural heteroscedasticity with OLS to estimate model (2).

#### Instrumental variable approach

When examining the effects of health insurance on self-protection behaviors, the endogeneity of insurance participation should be considered, otherwise the estimate would be biased. Theoretically, combined with the research content of this paper, the endogeneity stems from two main sources: (i) unobserved factors (for instance, risk aversion, health preference, etc.) may simultaneously affect an individual's decision of insurance participation and self-protection behaviors. Omitting these factors could lead to biased estimates when studying the effect of health insurance on self-protection behaviors of enrollees. For example, while people with higher risk aversion are more likely to enroll in health insurance, they are also more likely to invest more in self-protection (such as smoke and drink less, spend more on PME). (ii) There may be a reverse causality relationship between insurance participation and self-protection behaviors which can also lead to estimation bias.

In this paper, we use the IV method to address the endogeneity of insurance participation, and adopt two-stage least squares (2SLS) for the estimation.


(3) $$\begin{array}{l} insurance_{it}=\beta_{0}+\beta_{1}county_{it}+\beta_{2}X_{it}+\varepsilon_{it} \end{array}$$

(4) $$\begin{array}{l} y_{it}=\gamma_{0}+\gamma_{1}\hat{insurance_{it}}+\gamma_{2}X_{it}+\varepsilon_{it} \end{array}$$

Models (3) and (4), respectively, represent the first and the second-stage regressions in 2SLS. A valid instrument must meet two conditions: the power condition and the exclusion restriction condition (37). Following previous studies (38–41), we use *county*_*it*_ which denotes the health insurance participation rate of an individual's county in year *t* as *county*_*it*_ is highly correlated with one's participation in health insurance (*insurance*_*it*_) and does not directly influence one's self-protection behaviors.

## Results

### Descriptive analysis

Table 2 reports the descriptive statistics (mean and standard deviation) of the sample data. 68.8% of rural residents have health insurance coverage in the survey year. In terms of self-protection behaviors, the percentage of respondents that smoke, drink liquor, and have general obesity is, respectively, 30.6%, 26.9%, and 8.5%. The respondents have an average sedentary time of 2.551 h per day, an average BMI of 23.139. In terms of control variables, the average age of the total sample is 48.444 years, of which 48.3% are male, 83.0% belong to Han ethnic group, 88.5% have a spouse living with, 13.2% get sick in the past 4 weeks, 77.7% are presently working.

**Table 2:** Descriptive statistics.

**Variable**	**Mean/%**	**S.D**.	**Variable**	**Mean/%**	**S.D**.
**Independent variable**			Age^2^/100	25.366	13.657
Health insurance	0.688	0.463	Gender	0.483	0.500
**Dependent variables**			Ethnic	0.830	0.376
Smoking	0.306	0.461	Marriage	0.885	0.319
Drinking	0.269	0.443	Education (%)		
Sedentary	2.551	1.894	Primary school and below	50.096%	
PME	1.158	1.919	Junior high school	38.617%	
BMI	23.139	3.508	Senior high school and above	11.287%	
Obesity	0.085	0.279	Health status	0.132	0.338
**Control variables**			Income	8.600	2.038
Age	48.444	13.778	Working	0.777	0.417

### Benchmark regression results

Table 3 reports the empirical results of the effects of health insurance on self-protection behaviors. The dependent variables in columns (1)–(6) are smoking, drinking, sedentary, PME, BMI, and obesity, respectively. In particular, columns (1), (2), and (6) are the probit estimation results with control variables included, columns (3)–(5) are the OLS estimation results with control variables added. The effects of health insurance on an individual's healthy lifestyle are revealed by column (1)–(3) and the results show that the effects of health insurance on smoking, drinking, and sedentary are not significant, which can be preliminarily inferred that people' healthy lifestyle was not changed by enrolling in health insurance. In terms of preventive medical usage, the result in column (4) shows that participating in health insurance significantly decreases one's PME (coefficient −0.966, *p* < 0.01). In terms of obesity risk, the results in columns (5) and (6) show that participating in health insurance exerts a significant effect on one's BMI (coefficient 0.493, *p* < 0.01), and increases the probability of having general obesity (coefficient 0.022, *p* < 0.01), which means that health insurance participation may increase the obesity risk of rural residents. However, whether the above results are reliable still needs to be further tested as there might be endogenous problems in the benchmark model.

**Table 3:** Benchmark regression results (Probit and OLS).

**Variable**	**(1)**	**(2)**	**(3)**	**(4)**	**(5)**	**(6)**
	**Smoking**	**Drinking**	**Sedentary**	**PME**	**BMI**	**Obesity**
Health insurance	−0.014	0.011	0.028	−0.966***	0.493***	0.022***
	(0.009)	(0.009)	(0.045)	(0.278)	(0.077)	(0.007)
Age	0.008***	0.016***	−0.057***	−0.141***	0.247***	0.008***
	(0.001)	(0.001)	(0.007)	(0.035)	(0.012)	(0.001)
Age^2^/100	−0.008***	−0.014***	0.039***	0.101***	−0.254***	−0.008***
	(0.001)	(0.001)	(0.007)	(0.031)	(0.012)	(0.001)
Gender	0.470***	0.407***	0.186***	0.308*	−0.368***	−0.024***
	(0.003)	(0.004)	(0.029)	(0.183)	(0.053)	(0.004)
Ethnic	−0.023***	−0.066***	−0.179***	−0.441	0.574***	0.023***
	(0.007)	(0.007)	(0.038)	(0.314)	(0.066)	(0.006)
Marriage	0.003	0.012	−0.225***	0.106	0.374***	0.019**
	(0.009)	(0.010)	(0.053)	(0.223)	(0.088)	(0.008)
Junior high school	−0.021***	0.001	0.150***	0.230	0.147**	0.001
	(0.006)	(0.006)	(0.033)	(0.213)	(0.060)	(0.005)
Senior high school and above	−0.034***	0.007	0.482***	0.907***	0.136	−0.008
	(0.008)	(0.009)	(0.053)	(0.331)	(0.089)	(0.007)
Health status	−0.007	−0.008	0.117***	0.893***	−0.105	0.006
	(0.008)	(0.008)	(0.041)	(0.206)	(0.078)	(0.006)
Income	−0.005***	0.001	−0.007	−0.001	0.025*	0.000
	(0.001)	(0.001)	(0.008)	(0.054)	(0.013)	(0.001)
Working	0.006	0.023***	−0.374***	−0.123	−0.363***	−0.017***
	(0.008)	(0.008)	(0.043)	(0.202)	(0.070)	(0.005)
Year dummy variables	Yes	Yes	Yes	Yes	Yes	Yes
Observations	19,221	19,243	17,610	438	18,014	18,014
*R*-squared	–	–	0.064	0.262	0.062	–

Standard errors are in parentheses; *p < 0.1, **p < 0.05, ***p < 0.01. In columns (1), (2), and (6), the statistics reported are the marginal effects of independent variables.

### Instrumental variable regression results

As discussed above, potential endogeneity can lead to estimation bias. This paper uses IV to address the endogeneity of health insurance, and Table 4 reports the results. From the regression results of the first stage, the coefficients of IV are all positive at the 1% significant level, indicating that there is a strong correlation between our IV and endogenous dependent variable. Additionally, the *F*-values in the first stage regression are > 10, indicating that there is no weak IV problem, and the IV used in our model is valid (42). The results of the second stage regression show that: First, from perspective of healthy lifestyle, health insurance exerts no significant effect on smoking and sedentary, while participating in health insurance increases an individual's probability of drinking liquor by 3.4% at the 5% significance level, as shown in columns (1)–(3). Second, in terms of preventive medical usage, the result in column (4) shows that health insurance participation reduces people's PME by 1.057%, at the 1% significance level. Third, the result in columns (5) and (6) shows that health insurance enrollment significantly increases one's BMI by 0.784 kg/m^2^ (at the 1% level), and participating in health insurance increases enrollee' propensity toward having general obesity by 3.7% at the 1% significance level, indicating that health insurance participation may increase the obesity risk of rural residents. These findings reveal that there does exist ex ante moral hazard induced by health insurance in rural China.

**Table 4:** Instrumental variable regression results.

**Variable**	**(1)**	**(2)**	**(3)**	**(4)**	**(5)**	**(6)**
	**Smoking**	**Drinking**	**Sedentary**	**PME**	**BMI**	**Obesity**
**Part A: The first stage**					
IV	1.011***	1.012***	1.019***	0.961***	1.015***	1.015***
	(0.007)	(0.007)	(0.008)	(0.056)	(0.008)	(0.007)
First stage *F*-value	5139.84	5142.68	17809.70	305.77	17713.15	4760.92
Control	Yes	Yes	Yes	Yes	Yes	Yes
**Part B: The second stage**					
Health insurance	0.007	0.034**	0.023	−1.057***	0.784***	0.037***
	(0.015)	(0.013)	(0.053)	(0.373)	(0.104)	(0.008)
Control	Yes	Yes	Yes	Yes	Yes	Yes
Observations	19,221	19,243	17,610	438	18,014	18,014

Standard errors are in parentheses; *p < 0.1, **p < 0.05, ***p < 0.01. In columns (1), (2), and (6), the statistics reported are the marginal effects of independent variables.

Comparing regression results in Tables 3, 4, it can be seen that in the benchmark model, there is no significant effect on people's tendency to drinking, while after using the IV method to control the endogeneity, the marginal effect of health insurance becomes significant (coefficient 0.034, *p* < 0.05). In addition, the effect of health insurance on PME (coefficient −1.057), BMI (coefficient 0.784), and having general obesity (coefficient 0.037) in IV estimation is larger than that in the benchmark model (−0.966, 0.493, 0.022, respectively). The above difference suggests that ignoring endogeneity problems would lead to underestimation of the negative impact of health insurance on self-protection. One possible reason for such difference may be that the unobserved factors (such as risk preference) which could affect both the health insurance participation decision and the self-protection decision are ignored in benchmark model. As people with high risk-aversion are both more likely to enroll in health insurance and to have more self-protective behaviors (such as avoid drinking, spend more on PME), ignoring such factors tend to bias the impact downwards.

### Heterogeneity analysis

Although the above analyses estimate the average effect of health insurance participation on residents' self-protection behaviors, we do not take the potential differences among different groups of the insured into consideration. From the perspective of identifying ex ante moral hazard caused by health insurance more accurately, and then promoting the optimization of health insurance, it is more practical and significant to investigate which group is more affected by the policy (43). Therefore, it is necessary to examine the heterogeneity of health insurance's effect. To further examine the influences of health insurance participation on self-protection behaviors of different groups, we analyse the heterogeneity by gender, age, and educational level. Similarly, in order to address the endogeneity, the heterogeneity analyses below are all based on IV method.

Table 5 shows the results of heterogeneity by gender. In terms of healthy lifestyle, health insurance enrollment exerts a larger effect on drinking among males (coefficient 0.040, *p* < 0.1) than females (coefficient 0.019, *p* < 0.05). In terms of preventive medical usage, participating health insurance exerts no significant effect on PME of male residents, but significantly decreases that of female residents by 1.272% at the 1% significance level. In terms of obesity risk, after participating in health insurance, the BMI of male and female residents significantly increases by 0.649 and 0.868 kg/m^2^, respectively, and the likelihood of having general obesity of male and female also significantly increases by 3.1% and 3.4%, respectively.

**Table 5:** Heterogeneity by gender.

**Variable**	**Smoking**		**Drinking**		**Sedentary**	
	**Male**	**Female**	**Male**	**Female**	**Male**	**Female**
Health insurance	0.014	−0.003	0.040*	0.019**	0.073	−0.026
	(0.024)	(0.008)	(0.024)	(0.007)	(0.081)	(0.070)
Control	Yes	Yes	Yes	Yes	Yes	Yes
First stage *F*-value	2656.49	2849.23	2655.73	2853.13	9118.38	10091.37
Observations	9,280	9,941	9,297	9,946	8,564	9,046
**Variable**	**PME**		**BMI**		**Obesity**	
	**Male**	**Female**	**Male**	**Female**	**Male**	**Female**
Health insurance	−0.847	−1.272***	0.649***	0.868***	0.031***	0.041***
	(0.703)	(0.440)	(0.145)	(0.147)	(0.011)	(0.012)
Control	Yes	Yes	Yes	Yes	Yes	Yes
First stage *F*-value	261.21	177.43	8875.31	10238.74	2412.24	2687.43
Observations	161	277	8,567	9,447	8,567	9,447

Standard errors are in parentheses; *p < 0.1, **p < 0.05, ***p < 0.01. Variable age and its square are not included in control variables. When independent variables are smoking, drinking, and obesity, the statistics reported are the marginal effects.

Table 6 reports the results of heterogeneity by age. The whole sample is divided into two age groups: young (18–60 years old) and old (60 years old and above). First, participating in health insurance significantly increases the probability of drinking liquor of young rural residents with a marginal effect of 3.9%, but has no significant effect on that of old rural residents. Second, the effect of health insurance on PME is larger and more significant among young rural residents (coefficient −1.127, *p* < 0.05) than old rural residents (coefficient −0.133, non-significant). Third, enrolling in health insurance can significant increase young (old) residents' BMI and tendency to having general obesity by 0.714 (1.112) kg/m^2^ and 3.8 (3.4)%, respectively.

**Table 6:** Heterogeneity by age.

**Variable**	**Smoking**		**Drinking**		**Sedentary**	
	**Young**	**Old**	**Young**	**Old**	**Young**	**Old**
	**(18–60)**	**(60 and above)**	**(18–60)**	**(60 and above)**	**(18–60)**	**(60 and above)**
Health insurance	0.002	0.026	0.039***	0.007	0.007	0.180
	(0.016)	(0.036)	(0.014)	(0.035)	(0.058)	(0.127)
Control	Yes	Yes	Yes	Yes	Yes	Yes
First stage *F*-value	4042.15	1017.88	4043.06	1019.65	14255.00	3105.61
Observations	14,958	4,263	14,975	4,268	13,950	3,660
**Variable**	**PME**		**BMI**		**Obesity**	
	**Young**	**Old**	**Young**	**Old**	**Young**	**Old**
	**(18–60)**	**(60 and above)**	**(18–60)**	**(60 and above)**	**(18–60)**	**(60 and above)**
Health insurance	−1.127**	−0.133	0.714***	1.112***	0.038***	0.034*
	(0.477)	(0.483)	(0.113)	(0.256)	(0.010)	(0.015)
Control	Yes	Yes	Yes	Yes	Yes	Yes
First stage *F*-value	195.15	52.03	14020.99	3270.01	3707.97	987.81
Observations	284	154	13,917	4,097	13,917	4,097

Standard errors are in parentheses; *p < 0.1, **p < 0.05, ***p < 0.01. When independent variables are smoking, drinking, and obesity, the statistics reported are the marginal effects.

Table 7 shows the results of heterogeneity by educational level. Similarly, the total sample is classified into two groups: low (educational level is primary school and below) and high (junior high school and above). In terms of healthy lifestyle, the effects of health insurance on smoking are not significant in both groups; health insurance participation only significantly increases one's tendency to drinking among low-educated residents by 4.7%; in addition, after participating health insurance, people with low education tend to spend less time on sedentary activities while people with high education tend to be more sedentary. In terms of preventive medical usage, the effect is larger and more significant among high-educated residents (coefficient −1.978, *p* < 0.01) than low-educated residents (coefficient −0.274, non-significant). Finally, in terms of obesity risk, after participating in health insurance, the BMI, and probability of having general obesity for high-educated (low-educated) residents significantly increase by 0.761 (0.770) kg/m^2^ and 4.9 (2.4)%, respectively.

**Table 7:** Heterogeneity by educational level.

**Variable**	**Smoking**		**Drinking**		**Sedentary**	
	**Low**	**High**	**Low**	**High**	**Low**	**High**
Health insurance	0.022	−0.015	0.047***	0.018	–0.110*	0.150*
	(0.019)	(0.023)	(0.017)	(0.021)	(0.063)	(0.086)
Control	Yes	Yes	Yes	Yes	Yes	Yes
First stage *F*-value	3095.87	2837.48	3101.37	2835.73	11863.32	9214.23
Observations	9,629	9,592	9,640	9,603	8,569	9,041
**Variable**	**PME**		**BMI**		**Obesity**	
	**Low**	**High**	**Low**	**High**	**Low**	**High**
Health insurance	–0.274	–1.978***	0.770***	0.761***	0.024*	0.049***
	(0.319)	(0.672)	(0.143)	(0.151)	(0.012)	(0.011)
Control	Yes	Yes	Yes	Yes	Yes	Yes
First stage *F*-value	151.50	190.41	11854.33	8975.56	2916.07	2583.32
Observations	229	209	9,162	8,852	9,162	8,852

Standard errors are in parentheses; *p < 0.1, **p < 0.05, ***p < 0.01. Education-related variables are not included in control variables. When independent variables are smoking, drinking, and obesity, the statistics reported are the marginal effects.

### Robustness check

In addition to the above main results, we also conduct following robustness checks to verify the robustness of previous conclusions. First, in this study, there are outliers in our control variable *Income*. Hence, in order to further eliminate the effect of outliers, we winsorize the variable *Income* at the top and bottom 5% (Table 8). Second, since previous studies on ex ante moral hazard induced by health insurance used CHNS data before 2011 (31, 32), we also restrict the sample period from 2004 to 2011 and 2004 to 2009, respectively (Table 9). Third, we adopt stepwise regression method to test the robustness through adding control variables (population characteristics and health status, socioeconomic status, the year of interview) step by step (Table 10). Finally, we apply different estimation method to check the robustness (Table 11). Specifically, IV-regress estimator is used to estimate the effect of health insurance on smoking, drinking, and having general obesity, while Generalized Method of Moments (GMM) is adopted to estimate the effect of health insurance on sedentary, PME, and BMI. As expected, the significance and sign of estimators in above robustness test remain consistent with those in Table 4. Therefore, the findings in this paper are robust.

**Table 8:** Robustness check 1.

**Variable**	**(1)**	**(2)**	**(3)**	**(4)**	**(5)**	**(6)**
	**Smoking**	**Drinking**	**Sedentary**	**PME**	**BMI**	**Obesity**
Health insurance	0.006	0.037***	0.032	−1.268***	0.762***	0.034***
	(0.015)	(0.014)	(0.054)	(0.400)	(0.104)	(0.009)
Control	Yes	Yes	Yes	Yes	Yes	Yes
First stage *F*-value	4569.66	4571.49	15892.59	222.08	14879.87	4255.80
Observations	17,298	17,319	15,881	392	14,788	16,219

Standard errors are in parentheses; *p < 0.1, **p < 0.05, ***p < 0.01. When independent variables are smoking, drinking, and obesity, the statistics reported are the marginal effects.

**Table 9:** Robustness check 2.

**Variable**	**(1)**	**(2)**	**(3)**	**(4)**	**(5)**	**(6)**
	**Smoking**	**Drinking**	**Sedentary**	**PME**	**BMI**	**Obesity**
**2004–2011**						
Health insurance	0.008	0.033**	0.014	−1.066***	0.762***	0.032***
	(0.015)	(0.014)	(0.052)	(0.387)	(0.104)	(0.008)
Control	Yes	Yes	Yes	Yes	Yes	Yes
First stage *F*-value	4347.17	4354.69	14770.23	314.28	14879.87	4097.53
Observations	15,678	15,696	14,375	330	14,788	14,788
**2004–2009**						
Health insurance	0.009	0.035**	0.014	−1.089***	0.787***	0.032***
	(0.016)	(0.014)	(0.052)	(0.382)	(0.104)	(0.008)
Control	Yes	Yes	Yes	Yes	Yes	Yes
First stage *F*-value	3191.99	3199.11	8742.14	137.00	8622.56	3001.86
Observations	12,012	12,030	10,888	212	11,264	11,264

Standard errors are in parentheses; *p < 0.1, **p < 0.05, ***p < 0.01. When independent variables are smoking, drinking, and obesity, the statistics reported are the marginal effects.

**Table 10:** Robustness check 3.

**Variable**	**Smoking**		**Drinking**		**Sedentary**	
Health insurance	0.002	0.004	0.030**	0.036***	−0.081	0.000
	(0.015)	(0.015)	(0.014)	(0.013)	(0.054)	(0.053)
Demographic characteristics and health status	No	Yes	No	Yes	No	Yes
Socioeconomic characteristics	No	No	No	No	No	No
The year of interview	Yes	Yes	Yes	Yes	Yes	Yes
The first stage *F*-value	15371.87	7007.61	15380.98	7011.62	50497.09	23978.90
Observations	19,221	19,221	19,243	19,243	17,610	17,610
**Variable**	**Preventive spending**		**Overweight**		**Obesity**	
Health insurance	−1.747***	−1.126***	0.912***	0.777***	0.041***	0.036***
	(0.423)	(0.388)	(0.106)	(0.104)	(0.008)	(0.008)
Demographic characteristics and health status	No	Yes	No	Yes	No	Yes
Socioeconomic characteristics	No	No	No	No	No	No
The year of interview	Yes	Yes	Yes	Yes	Yes	Yes
The first stage *F*-value	649.86	365.45	50328.72	23922.12	14239.05	6490.61
Observations	438	438	18,014	18,014	18,014	18,014

Standard errors are in parentheses; *p < 0.1, **p < 0.05, ***p < 0.01. When independent variables are smoking, drinking, and obesity, the statistics reported are the marginal effects.

**Table 11:** Robustness check 4.

**Variable**	**(1)**	**(2)**	**(3)**	**(4)**	**(5)**	**(6)**
	**Smoking**	**Drinking**	**Sedentary**	**PME**	**BMI**	**Obesity**
Health insurance	0.006	0.029**	0.023	−1.057***	0.784***	0.034***
	(0.012)	(0.012)	(0.053)	(0.373)	(0.104)	(0.008)
Control	Yes	Yes	Yes	Yes	Yes	Yes
First stage *F*-value	19492.12	19477.36	17809.70	305.77	17713.15	17713.15
Observations	19,221	19,243	17,610	438	18,014	18,014

Standard errors are in parentheses; *p < 0.1, **p < 0.05, ***p < 0.01. When independent variables are smoking, drinking, and obesity, the statistics reported are the marginal effects.

## Discussion

Theoretically, health insurance reduces the marginal gain of self-protection by narrowing the utility difference between healthy state and sick state, thereby leading to the decline of an individual's investment in self-protection. Based on the data from CHNS (2004–2015), this paper attempts to provide empirical evidence for the substitutional relationship between health insurance and self-protection, which means that there exists ex ante moral hazard induced by health insurance. Compared to existing research, we measure self-protection behaviors in a more comprehensive way. For instance, smoking, drinking, obesity are commonly used to measure people's self-protection behaviors (15, 19, 26, 27), but few studies have taken disease prevention (such as preventive medical usage) which is also an important form of self-protection into consideration. In this study, we not only use indicators that reflect an individual's healthy lifestyle and obesity risk as previous studies did, but also select PME to capture people's efforts to prevent diseases. Moreover, among few empirical studies on ex ante moral hazard caused by health insurance, little attention was paid to the heterogeneous influences of health insurance. For the minority literature that explored the heterogeneity of health insurance's effect, group heterogeneity is limited to gender (31, 32).

Altogether, this paper finds that health insurance exerts significantly negative effects on self-protection behaviors of the insured, and the effects of health insurance differ among different groups (gender, age, and educational level). First, the results above shows that health insurance participation increases the probability of drinking liquor among Chinese rural residents, but does not change people's propensity toward smoking. Similarly, Dave and Kaestner find that health insurance leads to an increase in the likelihood of drinking among elderly men in the United States (18), Peng and Qin find that rural residents covered by health insurance are more likely to engage in drinking (31). Considering that it is difficult to monitor whether and how much liquor is consumed by the insured in reality, we propose to suppress such moral hazard from two aspects. For one thing, the co-payment rate for diseases caused by excessive alcohol intake could be risen to increase the cost of drinking for the insured. In China, the co-payment rate of health insurance has not been differentiated for such diseases, so the differentiated co-payment rate would be an option worth trying. For another thing, the popularization of health knowledge could be enhanced to guide the insured to live a healthy lifestyle and avoid excessive drinking.

Second, this study finds that health insurance participation has a significant negative impact on rural residents' PME, which is contrary to the conclusions drawn by previous studies based on health insurance of USA and UK (14, 23). The main reason for this difference is that the coverage items of health insurance in China are different from those in developed countries. Specifically, health insurance in developed countries covers not only curative medical services but also preventive medical services, while health insurance in China only covers the former so far. Therefore, Chinese rural residents tend to replace non-reimbursable preventive medical services with reimbursed curative medical services, which may not only lead to rapid expansion of total medical expenditure, but also do harm to the long-term health of the insured. These consequences obviously deviate from the original objective of setting up health insurance to improve people's health. Considering the disease characteristics of Chinese rural residents recently, the disease prevention function of health insurance should be gradually improved, and more self-protection investment (such as screenings for cancer, cerebrovascular disease, heart disease, hypertension, etc.) could be included in the reimbursement scope in further reforms of health insurance.

Third, we also find that health insurance participation significantly increases one's obesity risk, as residents covered by health insurance have higher BMI and a higher probability of having general obesity. Similar findings are found in studies by Stanciole and Bhattacharya et al. (15, 19). Obesity risk is often considered as an indicator that can comprehensively reflect an individual's healthful diet and physical activity status, and people with a higher risk of obesity generally have a higher prevalence (32, 44, 45). This result suggests that participating in health insurance may exert negative impact on one's diet and exercise that are not discussed in this paper. This also enlightens that health insurance can provide some dietary knowledge and exercise courses for the insured for free to mitigate the negative impact of ex ante moral hazard.

In addition, there is group heterogeneity in the effect of health insurance on self-protection behaviors. Specifically, gender heterogeneity is mainly reflected in the usage of preventive medical care. Participating in health insurance inhibits the PME of females, but does not have a significant impact on that of males. This may be due to the fact that in rural China, men are the main labor force engaged in agricultural production, their opportunity cost of getting sick is higher than that of females, thereby males' health status gets more attention from family members (46). This means that in rural China, female residents may face a more serious depression in demand for preventive medical care and it is necessary to provide more policy supports for females. In the next phase of health insurance system reform, priority should be given to reducing the co-payment rate of preventive healthcare services for females, and screening items for high-risk diseases (such as breast cancer) could be included into the reimbursement scope as well.

Moreover, there are age-specific influences of health insurance participation on an individual's healthy lifestyle and PME as young rural residents are more likely to drink liquor and reduce their PME after getting insured, while health insurance participation does not present substantial impact on that of old rural residents. On the one hand, the elderly have lower health stock than the young (36). This fact makes the elderly more concerned about the changes in their health stock than the young. Therefore, even if health insurance brings lower prices for medical services, the elderly will not reduce their investment in self-protection. On the other hand, compared with the young, the elderly have more frequent contact with doctors due to their health problems, which makes the elderly have stronger health awareness. This implies that we should pay more attention to the publicity of health knowledge among young and middle-aged groups in rural areas.

In terms of heterogeneity in education level, when covered by health insurance, low-educated residents are more likely to drink liquor, high-educated residents tend to spend more time on sedentary activities and decrease their PME, which reveals that educational heterogeneity should be considered in addressing ex ante moral hazard. For rural residents with low education, the dangers of excessive drinking should be well-informed. For rural residents with high education, more attention should be paid to publicizing the harm of sedentary and the benefits of preventive health care.

Since 2016, Chinese government has started the integration of basic health insurance for rural and urban residents, which means that the NRCMS covering rural residents and the Urban Resident Basic Medical Insurance Scheme (URBMS) covering urban residents would be merged into the Urban Rural Resident Basic Medical Insurance (URRBMI). The integration is almost complete in 2020. For rural residents, the main difference between the NRCMS and the URRBMI is that the URRBMI provides lower co-payment rate of healthcare services, thereby the price of healthcare services is further reduced. According to the theoretical model and empirical analysis above, it can be inferred that ex ante moral hazard in the URRBMI would become more serious and prevalent.

## Conclusion

Based on the above results, this study draws the following conclusions. In a word, participation in health insurance significantly reduces the demand for self-protection of the insured. Specifically, after controlling for endogeneity, health insurance participation significantly increases people's tendency to drinking and having general obesity, as well as their BMI, and decreases their expenditures on preventive medical care, suggesting that ex ante moral hazard is also present in rural China. Further analysis shows that there is heterogeneity between groups. In particular, health insurance participation reduces PME of rural residents who are female, younger, and high-educated, and increases the tendency toward drinking of rural residents who are younger and low-educated.

## Limitations

Although this paper has comprehensively investigated the effect of health insurance on self-protection, there are several limitations. First, although the measurement of self-protection behaviors in this paper involves six indicators in three aspects: healthy lifestyle, preventive medicine usage, and obesity risk, we cannot fully describe all self-protection behaviors of the insured due to the limitations of survey data. Second, as the findings in this paper are based on the sample of rural residents, it requires caution when generalizing our conclusions to the Basic Medical Insurance Systems for Urban and Rural Residents which has been implemented in all provinces in China since 2020. Third, limited by the survey data, this paper only conducts a theoretical analysis on the mechanism of health insurance affecting self-protection, but does not provide any empirical evidences. Nonetheless, the above shortcomings may provide some inspiration and insights for future research.

## Data availability statement

Publicly available datasets were analyzed in this study. This data can be found here: https://www.cpc.unc.edu/projects/china.

## Ethics statement

Ethical review and approval was not required for the study on human participants in accordance with the local legislation and institutional requirements. Written informed consent for participation was not required for this study in accordance with the national legislation and the institutional requirements.

## Author contributions

YL conceived the study and wrote the first draft. LL helped in the writing of the final draft of the manuscript. JL proposed amendments. All authors contributed to the article and approved the submitted version.

## Funding

This study was supported by the National Social Science Foundation of China (16BSH046).

## Conflict of interest

The authors declare that the research was conducted in the absence of any commercial or financial relationships that could be construed as a potential conflict of interest.

## Publisher's note

All claims expressed in this article are solely those of the authors and do not necessarily represent those of their affiliated organizations, or those of the publisher, the editors and the reviewers. Any product that may be evaluated in this article, or claim that may be made by its manufacturer, is not guaranteed or endorsed by the publisher.
